# Beyond magnesium sulphate – Rethinking magnesium’s impact on maternal and foetal health in low to middle-income countries: A scoping review

**DOI:** 10.4102/safp.v68i1.6196

**Published:** 2026-01-13

**Authors:** Naeera Abdul, Vinogrin Dorsamy, Chauntelle Bagwandeen

**Affiliations:** 1 Department of Laboratory Medicine and Medical Sciences, Faculty of Health Sciences, University of KwaZulu-Natal, Durban, South Africa; 2 School of Public Health and Nursing, Faculty of Health Sciences, University of KwaZulu-Natal, Durban, South Africa

**Keywords:** hypertensive disorders of pregnancy, pre-eclampsia, magnesium, HIV, obesity, scoping review

## Abstract

**Background:**

Hypertensive disorders of pregnancy (HDP), contribute greatly to maternal and perinatal morbidity and mortality, particularly in low-and middle-income countries (LMICs), undermining progress towards reducing maternal mortality. While magnesium sulphate is established for seizure prophylaxis, magnesium’s physiological and epidemiological significance in HDPs is underexplored. This scoping review mapped current evidence on maternal magnesium homeostasis and HDP association.

**Methods:**

Electronic databases were searched for studies reporting maternal magnesium levels, magnesium physiology in pregnancy or, magnesium supplementation in HDP. Eleven studies met the eligibility inclusion criteria. Data was charted for study design, magnesium biomarkers, outcomes and contextual factors.

**Results:**

Observational evidence demonstrated lower magnesium levels among women with HDP against normotensive controls and linked low magnesium with adverse foetal outcomes. Mechanistic studies supported this, highlighting magnesium’s role in endothelial function, vascular tone regulation and oxidative stress. Randomised trials evaluating magnesium supplementation showed inconsistent findings and were influenced by variations in formulation, dosage, timing and, underlying nutritional status. Contextual factors, such as HIV, obesity, renal function, and socioeconomic disadvantage, impacted magnesium homeostasis and HDP risk, particularly in LMICs.

**Conclusion:**

Magnesium insufficiency may contribute to HDP and adverse perinatal outcomes in LMICs, though causal pathways remain unconfirmed. Improved biomarker standardisation, mechanistic studies, and targeted supplementation trials in high-risk or deficient populations are needed.

**Contribution:**

This review highlights key inconsistencies in magnesium measurement, identifies contextual modifiers relevant to LMICs, and outlines priority areas for future research.

## Introduction

The United Nations’ Sustainable Development Goal (SDG) 3 sets a target of reducing the global maternal mortality ratio to under 70 per 100 000 live births by 2030 (target 3.1).^1^ Yet, hypertensive disorders of pregnancy (HDP), most notably pre-eclampsia (PE), continue to drive a substantial share of the roughly 295 000 annual maternal deaths, with more than 90% occurring in low- to middle-income countries (LMICs), where health systems are often overstretched and antenatal care access uneven.^2,3^ Achieving SDG 3 therefore demands not only improved access to interventions such as magnesium sulphate (MgSO_4_) for seizure prophylaxis but also a deeper understanding of magnesium’s (Mg) broader physiological role in blood-vessel health and placental function.

Magnesium is an essential cofactor in over 300 enzymatic reactions, including those governing vascular tone, endothelial integrity, and oxidative-stress regulation.^4,5,6^ Low magnesium intake and deficiency have been linked to hypertension and endothelial dysfunction in non-pregnant populations, suggesting potential relevance for HDP.^7^ Magnesium sulphate is well established for seizure prophylaxis in severe PE and eclampsia, yet this therapeutic role has overshadowed broader investigations into magnesium’s preventive or mechanistic contributions earlier in pregnancy.^5^

The available literature on magnesium and HDP spans diverse study designs, including biochemical, epidemiological, and interventional work conducted across heterogeneous populations. However, findings are often inconsistent. Some studies report strong associations between low magnesium levels and HDP, while others find no relationship.^8,9,10^ Variations in magnesium measurement (serum vs. intracellular), supplementation dose and timing, and failure to account for comorbidities such as human immunodeficiency virus (HIV) infection, obesity, and socioeconomic deprivation further complicate interpretation.

Given this fragmented evidence base, a scoping review was warranted to comprehensively map existing research, identify consistencies and gaps, and clarify methodological and contextual limitations in the study of magnesium’s role in maternal health.

The purpose of this scoping review is therefore to synthesise and characterise current evidence on magnesium homeostasis during pregnancy and its relationship with hypertensive disorders and adverse perinatal outcomes, with a particular focus on LMIC settings.

### Objectives

This scoping review aimed to:

- Describe mechanisms of Mg absorption, distribution, renal handling, and placental transfer during pregnancy.
- Synthesise existing evidence linking maternal Mg insufficiency to HDP and adverse perinatal outcomes.
- Examine evidence from interventional studies of magnesium supplementation. Explore the influence of comorbidities such as HIV, obesity, and socioeconomic status, on magnesium metabolism and HDP risk. Identify knowledge gaps and methodological limitations to guide future research in LMICs.

## Research methods and design

### Framework and reporting

This review followed the Arksey and O’Malley^11^ methodological framework, enhanced by Levac et al.,^12^ and reported in accordance with the PRISMA-ScR (Preferred Reporting Items for Systematic Reviews and Meta-Analyses extension for Scoping Reviews) guidelines.^13^

### Research questions

The following research questions had been addressed:

- What is known about magnesium homeostasis during pregnancy?
- How is magnesium insufficiency associated with HDP and perinatal outcomes?
- How do HIV, obesity, and socioeconomic status modify these relationships?

### Eligibility criteria

Included studies were peer-reviewed, English-language, human studies (1988–2025) addressing:

- Magnesium homeostasis and metabolism during pregnancy.
- Associations between magnesium status and HDP, preterm birth, or foetal growth restriction.
- Magnesium supplementation during pregnancy.
- Interactions between magnesium and comorbidities (HIV, obesity, socioeconomic status).

Exclusion criteria: non-human studies (unless mechanistic), editorials, conference abstracts, case reports, and studies without measurable magnesium data.

### Information sources and search strategy

Searches were conducted in PubMed, Scopus, Web of Science, the Cochrane Library, and Google Scholar from February 1988 to September 2025. Keywords included ‘*magnesium homeostasis*’, ‘*magnesium supplementation*’, ‘*preeclampsia*’, ‘*gestational hypertension*’, ‘*fetal growth restriction*’, ‘*HIV*’, ‘*obesity*’, and ‘*socioeconomic status*’.

Reference lists of eligible articles were hand-searched for additional studies.

### Selection process

**FIGURE 1:**
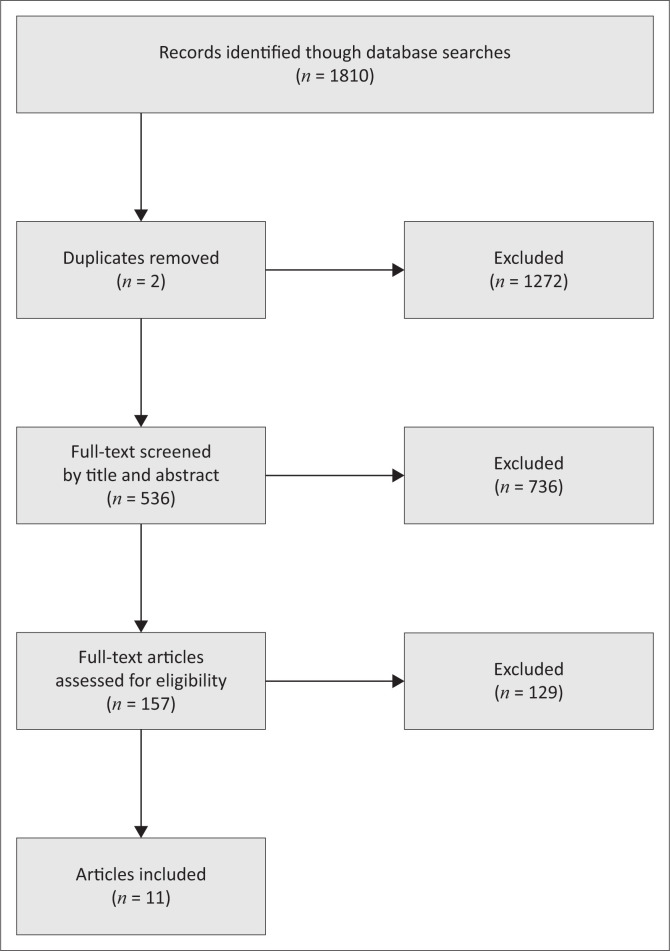
Preferred Reporting Items for Systematic Reviews and Meta-Analyses extension for Scoping Reviews flow diagram of study selection.

Citations were screened by title and abstract for relevance, followed by full-text review. The selection process is summarised in Figure 1.

### Data charting

Data were extracted into a table capturing:

- author and year
- study design and location
- sample characteristics
- magnesium measure (serum, erythrocyte, dietary intake)
- outcomes assessed (HDP, preterm birth, foetal growth restriction)
- key findings: Two tables summarise epidemiological associations (Table 1) and interventional evidence (Table 2).

**TABLE 1:** Epidemiological studies on magnesium status and hypertensive disorders of pregnancy.

Author and year of publication	Country or setting	Study design	Sample size (*n*)	Magnesium measure	Key findings
Abdul et al. (2025)^14^	South Africa	Cross-sectional or comparative	200 per group	Serum Mg	Lower Mg in EOPE and LOPE vs controls
Ephraim et al. (2014)^9^	Ghana	Case–control	380	Serum Mg	Hypomagnesaemia more common in PE and PIH
Nwogu et al. (2024)^15^	Nigeria	Case–control	150	Serum Mg	Significantly reduced Mg in severe PE
Kanagal et al. (2014)^16^	India	Case–control	120	Serum Mg & Ca	Lower Mg associated with PE and adverse outcomes
Tavana and Hosseinmirzaei (2013)^17^	Iran	Nested case–control	500 screened	Serum Mg	Lower Mg at baseline predicted PE development
Gupta (2020)^18^	India	Comparative	200	Serum Mg	Low Mg associated with LBW and PE
Tesfa et al. (2024)^19^	Ethiopia (meta-analysis)	Systematic review and meta-analysis	17 pooled studies	Serum Mg	SMD –1.20 mmol/L lower in PE vs controls

Note: Please see full reference list of Abdul N, Dorsamy V, Bagwandeen C. Beyond, magnesium sulphate – Rethinking magnesium’s impact on maternal and foetal health in low to middle-income countries: A scoping review. S Afr Fam Pract. 2025;67(1), a6196. https://doi.org/10.4102/safp.v67i1.6196

These findings highlight consistent reductions in magnesium concentrations among women with hypertensive disorders of pregnancy.

Mg, magnesium; EOPE, early-onset pre-eclampsia; LOPE, late-onset pre-eclampsia; PE, pre-eclampsia; PIH, pregnancy-induced hypertension; LBW, low birthweight; SMD, standardised mean difference.

**TABLE 2:** Interventional Studies on Magnesium Supplementation in Pregnancy.

Author and publication year	Country	Study design	Sample size (*n*)	Intervention	Comparator	Primary findings
De Araújo et al. (2020)^20^	Brazil	Randomised controlled trial	318	Mg citrate 300 mg/day	Placebo	No significant reduction in PE (18.1% vs. 19.7%)
Zarean and Tajan (2017)^21^	Iran	Randomised supplementation trial	200	Mg supplement (dose reported)	Standard care	Reduction in composite complications; ↑ birthweight
Khaity et al. (2022)^22^	Global	Meta-analysis of RCTs	11 trials	Various Mg formulations	Placebo/none	Pooled OR 0.77 (trend towards benefit)
Makrides et al. (2014)^23^	International	Cochrane systematic review	Multiple RCTs	Various Mg supplements	Placebo/none	Evidence insufficient for PE prevention
Kusuma et al. (2024)^24^	Indonesia	Therapeutic MgSO₄ kinetics study	80	MgSO₄ dosing	None	Describes pharmacokinetics; contextual rather than preventive

Note: Please see full reference list of Abdul N, Dorsamy V, Bagwandeen C. Beyond, magnesium sulphate – Rethinking magnesium’s impact on maternal and foetal health in low to middle-income countries: A scoping review. S Afr Fam Pract. 2025;67(1), a6196. https://doi.org/10.4102/safp.v67i1.6196

Across trials, the preventive effect of magnesium supplementation remains inconsistent, reflecting variation in baseline deficiency, formulation, and study quality.

Mg, magnesium; MgSO₄, magnesium sulphate; PE, Pre-eclampsia.

### Synthesis of results

Findings were synthesised narratively and thematically. No quantitative meta-analysis was attempted because of heterogeneity in study design, magnesium measures, and populations.

### Ethical considerations

Ethical clearance to conduct this study was obtained from the Biomedical Research Ethics Committee of the University of KwaZulu-Natal (No. BREC/00007005/2024). All samples were obtained with explicit informed consent. The purpose of the samples and tests were explained to all participants. All their data were kept private.

## Results

### Study selection and characteristics

A total of 11 studies met the inclusion criteria and were included in the final synthesis (Figure 1). These comprised observational studies evaluating the association between maternal magnesium status and HDP, as well as interventional trials assessing magnesium supplementation during pregnancy. The included studies were conducted across diverse global regions – with substantial representation from LMICs – and varied in design, sample size, magnesium measurement methods, and gestational timing of assessment.

### Epidemiological evidence of magnesium status and hypertensive disorders of pregnancy

Across observational and comparative studies, women who developed HDP consistently demonstrated lower circulating magnesium levels than normotensive controls. Studies from South Africa, Ghana, Nigeria, India, and Iran reported higher prevalence of hypomagnesaemia among women with PE and, in some cases, gestational hypertension. For example, Abdul et al.^14^ reported lower magnesium levels in both early-onset and late-onset PE, while Ephraim et al.^9^ and Nwogu et al.^15^ found significantly lower magnesium concentrations among women with PE compared with controls. Similarly, Kanagal et al.,^16^ Tavana and Hosseinmirzaei,^17^ and Gupta^18^ observed reduced serum magnesium levels in women with HDP. Meta-analytic evidence further supports this pattern. Tesfa et al.^19^ reported a pooled standardised mean difference of –1.20 mmol/L, indicating consistently lower magnesium among women with PE across multiple LMIC settings.^19^ A summary of key epidemiological studies is presented in Table 1.

### Inconsistencies and heterogeneity

Despite the overall consistency in direction of association, some variation exists between studies. Differences in laboratory assays, trimester of sampling, and cut-off thresholds for hypomagnesaemia contributed to inter-study variability.^20,21,22,23,24^ Additionally, few studies adjusted for important contextual modifiers such as HIV status, obesity, dietary intake, or socioeconomic status, which may independently influence magnesium homeostasis.^25,26,27,28,29^

### Interventional evidence: Magnesium supplementation studies

Evidence from intervention trials assessing magnesium supplementation for prevention of HDP remains mixed. In the largest Randomised Control Trial (RCT), De Araújo et al.^20^ found no significant difference in PE incidence between women receiving 300 mg/day of magnesium citrate and those receiving placebo. Conversely, smaller trials, such as Zarean and Tarjan,^21^ reported improvements in certain pregnancy outcomes, including birthweight and composite morbidity, although these studies were limited by small sample sizes and varied formulations.

Meta-analytic evidence suggests a possible trend towards reduced risk of HDP with supplementation, although the authors emphasised low certainty because of methodological limitations across trials.^22^ Similarly, the Cochrane review concluded that current evidence is insufficient to recommend routine magnesium supplementation for HDP prevention.^23^ The characteristics and findings of interventional studies are summarised in Table 2.

### Summary of findings

Overall, the evidence suggests that low magnesium status is frequently observed among women with HDP, particularly in LMIC settings where dietary magnesium intake may be inadequate. Observational findings demonstrate a consistent association between reduced magnesium levels and HDP, whereas interventional evidence is inconclusive and varies according to dosage, formulation, baseline deficiency, and study quality.

## Discussion

This scoping review synthesised evidence relating Mg status to HDP, integrating observational data, mechanistic studies, and interventional trials. Across diverse global settings, particularly within LMICs, a consistent pattern emerges linking lower maternal magnesium concentrations with PE and gestational hypertension. While the biological plausibility of this association is strong, limitations in measurement, heterogeneity of study designs, and inconsistent interventional evidence highlight the need for more focused, context-specific research.

### Magnesium homeostasis and physiological adaptations during pregnancy

Pregnancy induces major physiological shifts that influence magnesium distribution and metabolism. Several longitudinal studies report a progressive decline in serum magnesium from the second trimester onwards, driven by haemodilution, increased glomerular filtration, and foetal mineral requirements.^30,31,32,33^ Placental magnesium transport mechanisms, particularly through Transient Receptor Potential Melastatin (TRPM6) and (TRPM7) channels, are upregulated to safeguard foetal supply even when maternal levels decrease.^32^ These adaptations underscore the importance of maternal magnesium reserves for maintaining placental and foetal development.

Intracellular magnesium, reflected in erythrocyte levels, tends to remain more stable despite serum declines. However, in settings characterised by chronic dietary magnesium insufficiency – such as rural India, parts of sub-Saharan Africa, and other mineral-depleted regions – both serum and intracellular magnesium levels are consistently low.^16,18,32^ This indicates that physiological adaptations may be insufficient in nutritionally vulnerable populations, potentially increasing susceptibility to adverse pregnancy outcomes.

### Pathophysiological mechanisms linking magnesium to hypertensive disorders of pregnancy

There is compelling mechanistic evidence connecting low magnesium to the vascular and endothelial abnormalities characteristic of HDP. Magnesium plays a critical role in vascular smooth muscle relaxation, calcium homeostasis, nitric oxide synthesis, and modulation of oxidative stress^4,5,6^ Reduced magnesium availability promotes vasoconstriction, enhances vascular reactivity, and exacerbates endothelial dysfunction, central features in the pathogenesis of PE.^10,34,35,36^

Experimental data further demonstrates that impaired TRPM6-mediated magnesium transport results in placental and foetal defects resembling hypertensive pregnancy phenotypes.^37^ Moreover, women with low magnesium frequently exhibit higher levels of oxidative injury markers (e.g. malondialdehyde) and inflammatory cytokines such as Tumor Necrosis Factor (TNF)-α, along with reduced antioxidant capacity.^31^ These findings strengthen the biological plausibility that magnesium insufficiency could contribute to the pathophysiological cascade leading to HDP.

### Epidemiological associations and magnitude of effect

Epidemiological evidence consistently supports an association between lower magnesium levels and HDP. A meta-analysis focusing on African studies reported a pooled standardised mean difference of -1.20 mmol/L between women with PE and normotensive controls.^19^ Individual studies align with these findings: Tavana and Hosseinmirzaei^17^ reported significantly lower magnesium concentrations in Iranian women with PE, while a Nigerian study by Nwogu et al.^15^ documented even more pronounced reductions in severe cases. South African data from Abdul et al.^14^ showed similar patterns, including among early- and late-onset PE, with associations persisting after adjusting for HIV status and body mass index. These findings suggest that magnesium insufficiency may represent a consistent hallmark among women who develop HDP across a range of global contexts.

### Inconsistencies and heterogeneity across studies

Despite general consistency in the direction of association, notable heterogeneity limits cross-study comparability. Variability arises from several sources:

- **Magnesium biomarkers:** Studies measure total, ionised, or intracellular magnesium, each reflecting different physiological pools.
- **Assay methods and reference thresholds:** A lack of standardisation hinders comparison across populations.
- **Gestational age at sampling:** Measurements taken in different trimesters capture varying physiological states.
- **Dietary intake, renal function, and comorbidities:** Few studies adjust for these important modifiers.

Some studies measuring only total magnesium reported no significant differences, suggesting that serum magnesium may not fully capture biologically relevant magnesium status.^38,39^ Where multivariable adjustment was performed, effect sizes often strengthened, indicating that uncontrolled confounding may obscure true associations.

### Perinatal outcomes beyond blood pressure

Magnesium insufficiency has implications beyond maternal hypertensive disorders. Several cohort studies demonstrate associations between low maternal magnesium and adverse foetal outcomes, including small-for-gestational-age (SGA) infants, intrauterine growth restriction (IUGR), preterm birth, and low birthweight.^18,20,23,40^ Evidence from Brazil and India highlights a two-fold increased risk of low birthweight among women in the lowest magnesium tertiles.^18,20^ These findings are consistent with magnesium’s role in placental vasodilation, angiogenesis, and oxidative balance, suggesting that insufficiency may impair placental perfusion and foetal growth.^4,5,6,10,34,36^

### Interventional evidence: Magnesium supplementation studies

Interventional evidence is less clear than observational findings. Twelve randomised or quasi-randomised trials have assessed magnesium supplementation during pregnancy, but results vary widely. The largest trial, the BRAzilian MAGnesium Trial, reported no reduction in PE incidence with 300 mg/day magnesium citrate.^20^ Conversely, smaller Iranian trials demonstrated reduced PE incidence, IUGR, and preterm delivery among supplemented women.^21^ A meta-analysis of 11 RCTs suggested a non-significant protective trend (pooled Odds Ratio (OR) 0.77).^22^ Similarly, the 2014 Cochrane Review found that magnesium supplementation may reduce risks of low birthweight and preterm birth, but evidence remains insufficient to recommend supplementation for HDP prevention.^23^

Effectiveness appears influenced by contextual factors, including baseline magnesium deficiency, dietary patterns, supplement formulation, adherence, and gestational timing of initiation. Collectively, evidence indicates that targeted supplementation in deficient or high-risk populations may hold promise, whereas universal prophylaxis lacks sufficient support.

### Contextual modifiers: Human immunodeficiency virus (HIV), obesity, and socioeconomic status

#### Human immunodeficiency virus (HIV) infection and antiretroviral therapy

Human immunodeficiency virus infection may depress magnesium levels through chronic inflammation and altered renal handling, while antiretroviral therapy (ART), particularly protease inhibitors, may exacerbate endothelial dysfunction.^25,26,41^ South African data show that HIV-positive women had significantly lower magnesium levels and a higher risk of PE, although ART regimen influenced the magnitude of this association.^14^

#### Obesity and metabolic risk

Obesity is associated with reduced intracellular magnesium because of chronic inflammation and insulin resistance.^42^ Several studies document inverse correlations between Body Mass Index (BMI) and serum magnesium in pregnancy, suggesting that obese women may be particularly susceptible to the adverse vascular effects of magnesium deficiency.^28,29^

#### Socioeconomic and nutritional factors

The LMICs often face widespread dietary magnesium insufficiency linked to cereal-based diets, mineral-depleted soils, and limited dietary diversity.^43^ Structural challenges – such as late antenatal booking, stock-outs of MgSO_4_, and limited provider training – may further heighten the risk of HDP and maternal morbidity.

### Knowledge gaps identified

This article highlights several key gaps:

- Measurement variability: A lack of consensus on the most accurate magnesium biomarker.
- Inadequate deficiency thresholds: No pregnancy-specific cut-offs exist.
- Limited adjustment for confounders: Few studies account for HIV, obesity, diet, renal function, or socioeconomic status.
- Uncertain supplementation strategies: Optimal dosage, formulation, and timing remain unclear.
- Limited mechanistic integration: Few studies include endothelial, angiogenic, or oxidative biomarkers.
- Low- and middle-income countries evidence gap: Despite bearing the highest burden, LMIC-focused trials remain scarce.

### Limitations

This scoping review did not include formal quality appraisal of included studies, consistent with PRISMA-ScR guidelines. As a result, heterogeneity in study design, methodology, and measurement techniques limits direct comparisons. Most studies were observational and cross-sectional, restricting temporal inference. Confounding variables were inconsistently reported, and publication bias cannot be excluded. Despite these limitations, this review offers a comprehensive mapping of the available evidence and highlights priority areas for future research.

### Recommendations

Future research should prioritise standardisation of magnesium assessment, development of pregnancy-specific reference ranges, and use of more sensitive biomarkers such as ionised or intracellular magnesium. Longitudinal cohort studies are needed to clarify temporal relationships and better account for contextual modifiers, including HIV and obesity.

Large, well-powered randomised trials focused on women with documented magnesium deficiency are required to determine optimal dosage, formulation, and timing of supplementation. Trials should integrate mechanistic endpoints to clarify physiological effects.

In LMIC settings, strengthening antenatal nutritional assessment, promoting dietary diversification or fortification, and improving health system readiness, particularly reliable access to MgSO_4_, may enhance maternal outcomes.

## Conclusion

Taken together, the evidence reveals a biologically plausible and epidemiologically consistent association between magnesium insufficiency and HDP. While supplementation demonstrates potential benefit in deficient or high-risk populations, its preventive efficacy remains uncertain because of heterogeneity in trial design and population characteristics. Harmonised biomarker approaches, context-specific interventional research, and integration of mechanistic markers are essential to determine whether magnesium optimisation can contribute meaningfully to reducing the burden of HDP – particularly within LMICs, where both magnesium deficiency and HDP prevalence are high. This scoping review revealed a lack of magnesium reporting and investigation in RCTs, with comorbidities in LMIC-based studies being ignored and a scarcity of treatment protocols; thereby emphasising the importance of reinforcing treatment protocols and emphasising the importance of magnesium and how co-morbidities can affect it.
